# Does Heart Failure Mask Candida Tricuspid Endocarditis?

**DOI:** 10.7759/cureus.38951

**Published:** 2023-05-12

**Authors:** Anas Mahmoud, Alman B Khalid, Alejandra Ehle, Yusuf Kahf, Adil Afzal, Bradley Peltzer

**Affiliations:** 1 Internal Medicine, St. Joseph’s University Medical Center, Paterson, USA; 2 Cardiology, St. Joseph’s University Medical Center, Paterson, USA

**Keywords:** infective endocarditis, bioprosthetic valve, vegetation, candida, fungal endocarditis

## Abstract

Infective endocarditis (IE) carries high morbidity and mortality. Although minimal in incidence, fungal causes (mostly *Candida* species) carry the highest mortality among all cases of infective endocarditis. We describe a rare case of a 47-year-old male with a past medical history of cerebral vascular accident (CVA), heart failure with reduced ejection fraction status post (SP) automated implantable cardioverter defibrillator (AICD) placement, paroxysmal atrial fibrillation, coronary artery disease (CAD), infective endocarditis with mitral valve replacement and tricuspid valve replacement, and pulmonary hypertension who presented to the emergency department (ED) with complaints of shortness of breath and weakness for four days. The patient was admitted to the cardiac care unit (CCU) due to persistent hypotension despite being on a continuous milrinone drip at home. The patient was initially started on antimicrobial agents for sepsis most likely secondary to pneumonia. Echocardiographic imaging showed a large vegetation on the tricuspid valve; hence, blood cultures were sent and came back positive for *Candida *sp. Appropriate antifungals (micafungin) were added to the medication regimen, and the patient was transferred to a tertiary hospital for surgical intervention. Patients with bioprosthetic valve replacement require regular follow-ups as this would allow providers to catch symptoms of developing endocarditis and prevent disease progression. These appointments may also decrease other risk factors for the disease, including but not limited to infected lines.

## Introduction

Infection can occur in any of the three layers of the heart: the endocardium, myocardium, and pericardium. Endocardial infections are the most deleterious and carry the worst outcome, due to the fact that not only it does embolize to the brain and other organs but also the onset is very insidious and very difficult to anticipate and differentiate from other concomitant illnesses. On the other hand, the symptoms of pericarditis and myocarditis occur more rapidly and are relatively easier to diagnose. Patients with valve defects or prosthetic valves are more prone to develop endocardial infections due to bacterial adherence, which further damages the valves by creating holes and subsequently more bacterial adherence.

*Candida* infective endocarditis (CIE) is a rare subtype of infective endocarditis (IE) that is associated with serious complications and occurs more in patients with high-risk factors such as advanced age, prosthetic valves, history of recreational intravenous drug usage, and immunosuppression. Almost 90% of infective endocarditis causes are bacterial secondary to gram-positive *Streptococcus*, *Staphylococcus*, and *Enterococcus* infection. Of the remaining, fungal pathogens account for only 2% of infective endocarditis causes. Out of the total cases of fungal-caused infective endocarditis, *Candida* spp. are responsible for approximately 6% of them. Nonetheless, *Candida* infective endocarditis carries the highest mortality of all causes of infective endocarditis.

## Case presentation

**Figure 1:**
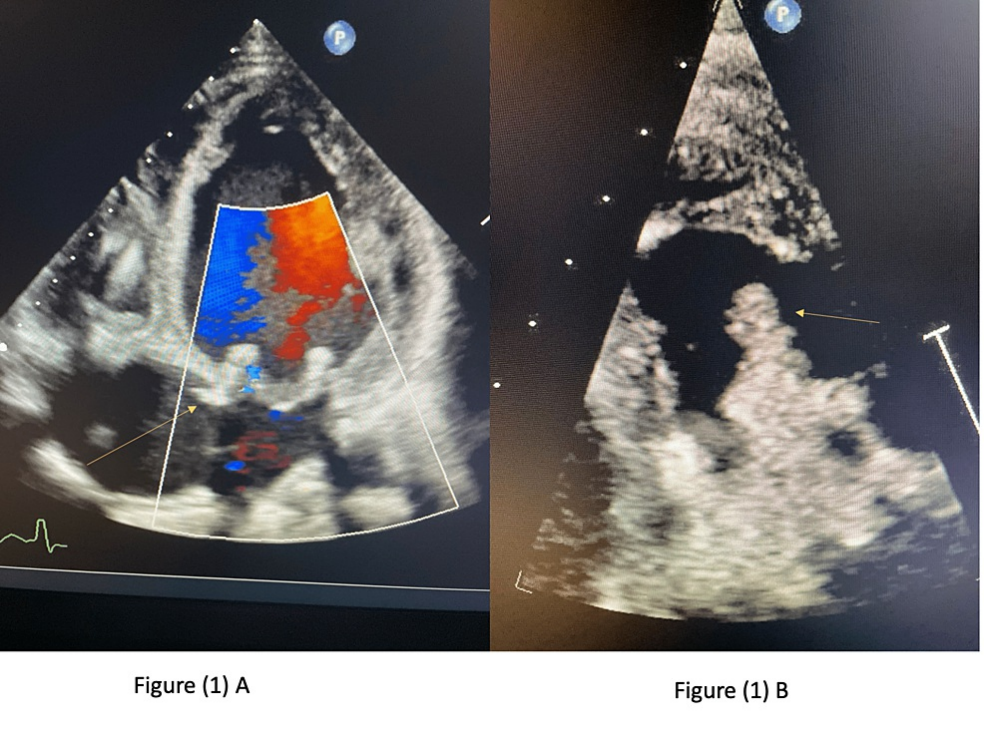
(A) Extensive vegetation causing severe tricuspid stenosis (yellow arrow). (B) Very big-sized vegetations on the tricuspid valve (yellow arrow)

A 47-year-old male with a past medical history of cerebral vascular accident (CVA) (2020), reduced ejection fraction heart failure status post (SP) automated implantable cardioverter defibrillator (AICD), paroxysmal atrial fibrillation, coronary artery disease (CAD), infective endocarditis SP mitral valve replacement (33 mm St. Jude Epic porcine valve {St. Jude Medical, Inc., St. Jude, MN}) and tricuspid valve replacement (tricuspid Hancock II porcine valve), and pulmonary hypertension presented to the emergency department (ED) for the evaluation of a four-day duration of dizziness and weakness, which increase with exertion. He stated that he had been experiencing consistent low blood pressures (80/60 mmHg), which was managed with a home milrinone drip. The patient denied any current illicit drug use or sick contact; however, on a previous admission, the patient presented with fever and septic shock-like picture and was found to have bacteremia secondary to *Staphylococcus lugdunensis* infection. Thus, he received a six-week course of daptomycin. On this admission, vitals upon arrival were significant only for hypotension. Workup in the ED revealed that the patient has severe anemia, signs of acute kidney injury (AKI), and lactic acidosis. Electrocardiogram (EKG) showed sinus rhythm, moderate intraventricular conduction delay, and a moderate T-wave abnormality. The cardiologist recommended admission to the cardiac care unit (CCU) for further observation and management due to the recurrent episodes of hypotension. In the CCU, the patient was started on antibiotics (ceftriaxone and doxycycline) for possible community-acquired pneumonia, given the worsening blood pressure and lactic acidosis. Meanwhile, a septic workup was sent and came back negative. Brain natriuretic peptide (BNP) level was noted to be elevated, while troponin came back normal. An echocardiogram (echo) showed extensive vegetation on the porcine tricuspid valve causing severe tricuspid stenosis (Figure 1), as well as the disruption of the posterior leaflet of the porcine mitral valve, though not due to endocarditis.

Cardiothoracic surgery was consulted for the evaluation of valves. Transesophageal echocardiogram (echo) was later obtained for a better visualization of the vegetation and valve function of the patient. Two sets of blood cultures were sent, and the patient was switched to vancomycin and piperacillin-tazobactam due to worsening leukocytosis despite afebrile status. Blood cultures came back positive and grew *Candida albicans*. Hence, the antibiotics were stopped, micafungin was started, and repeat blood cultures were sent with Fungitell Beta-D-Glucan assay (Associates of Cape Cod, Inc., Falmouth, MA), which came back elevated. Old peripherally inserted central catheter (PICC) line was replaced by a new one; then, the patient was transferred to a university hospital where the patient had a high-risk procedure done for the replacement of bioprosthetic tricuspid valve with the continuation of antifungals for a total of six weeks. Fortunately, the procedure was successful, and the patient tolerated it well and then was discharged to a rehabilitation center for a closer follow-up.

## Discussion

Endocarditis is an inflammation of the endothelial lining of the heart, which can be due to infection or noninfectious causes. Infective endocarditis (IE) is more common, and it is characterized by valve damage [1]. On the contrary, noninfective endocarditis (NIE) spares the valves, and they are not damaged. NIE is poorly understood; however, it is most likely caused by a combination of different interacting mechanisms: circulating immune complexes and carcinomatosis and depositing sterile fibrin and platelets around the leaflets, causing a hypercoagulable state around the valve, which does not destruct the valves. The common causes of NIE are either autoimmune disorder such as systemic lupus, any other autoimmune conditions, immunosuppressive agents, or malignancy treatment with chemotherapy or radiotherapy. As NIE is mainly due to a hypercoagulable state, anticoagulation is the mainstay of treatment, and surgery is seldom required due to the fact that the valves are spared and not damaged. Histology is mandatory for diagnosis as echo can not differentiate between IE and NIE.

IE is mainly caused by a pathogen, most commonly bacteria, which travels through the blood to the endothelial wall, attaches to the polysaccharide dextran, and forms a vegetation complex along with fibrin and platelets that triggers inflammation resulting in valve destruction. The vegetation results from rough blood movement across the endothelium lining the valves, making the fibronectin matrix nectin more resilient to penetration and eradication by antibiotics. Ongoing uncontrolled infection can progress to further tissue destruction including the conduction system, which can lead to heart failure or sudden cardiac arrest [2]. IE is mainly bacterial in origin, and rheumatic fever and mitral stenosis enhance innate valves to develop IE. Recently, with the high prevalence of prosthetic valves, the incidence of IE has increased. Indolent fever and leukocytosis can raise suspicion for IE, especially in intravenous drug users with innate valves or patients who replaced their valves before. Blood cultures are the mainstay of pathogen identification; however, some are difficult to grow in blood cultures because of their delicacy. Culture-positive IE (CPIE) represents 87% of all cases and includes *Streptococcus viridans* (23%); *Staphylococcus aureus* (22%); other *Streptococcus* species such as *S. bovis*, *S. beta-hemolyticus*, and *S. pneumococcus* (22%); and *Enterococcus* (10%). Culture-negative IE (CNIE) represents 13% and is further divided into infective endocarditis (10%) and NIE (3%). CNIE includes fungi and *Haemophilus*, *Aggregatibacter*, *Cardiobacterium*, *Eikenella*, and *Kingella* (HACEK) organisms, which might grow in special stains or culture media or regular blood culture less frequently.

Candidemia has been notoriously increasing in the United States with an annual incidence of 25000 cases per year. The spread of the infection to the heart is rare but unfortunately is associated with serious complications. *Candida* infective endocarditis (CIE) is a rare cause of IE; meanwhile, it is associated with a high mortality rate [3]. Rivoisy et al. studied the incidence of fungal infective endocarditis (FIE), and they found that 60% of FIE were found in patients who have bioprosthetic valves. Of those, only 13% were found to have vegetation on the tricuspid valve, while the highest infection rate was on the aortic (72%), making our findings of tricuspid valve involvement significantly scarce [4]. Patients with candidemia should be suspected for valvular infection, especially prosthetic valves, with persistent fever despite antifungals or any cardiac symptoms. Foong et al. [5] conducted a retrospective analysis on CIE and concluded that preexisting valvular heart disease was the only factor associated with CIE, while other causes such as total parenteral nutrition and hematologic malignancy were associated with lower risk. Therefore, invasive and expensive diagnostic cardiac imaging might not be indicated in lower-risk patients.

A great percentage of the high morbidity and mortality surrounding cases of fungal endocarditis and IE, in general, is due to the possible complications following infection [6]. Inpatient mortality rate was 16.2% in CIE. Liver failure (either acute or subacute) was associated with higher mortality; meanwhile, intravenous drug users have interestingly lower risk of death [6]. Comorbidities heavily impact these complications in these patients, and the early detection of the disease to begin treatment quickly could mitigate those complications [7]. The prolonged use of vascular lines, e.g., PICC line, might be a significant reason for the development of candidemia, although intravenous recreational drug use should be ruled out. CIE has also been reported in infants and is associated with high mortality; therefore, it is recommended to perform echocardiogram for any candidemia or positive *Candida* catheter culture [8].

Tricuspid valve pathologies often do not present with symptoms until the disease has progressed in severity. Symptomatic clues indicating severity include leg swelling, fatigue, and decreased exercise tolerance, correlating with the vegetation size [9]. Therefore, concurrent heart failure could mislead the diagnosis of tricuspid valve endocarditis due to similar presentation. CIE is treated via antifungals, often with azoles or echinocandins, as well as a surgical intervention [7]. Prompt diagnosis and comprehensive management are essential. Concurrent heart failure may delay the diagnosis and management, and the careful revision of the patient’s history can help overcome this problem.

## Conclusions

Heart failure could mask ongoing infective endocarditis since both conditions present with similar symptoms. Besides, some heart failure medications, such as milrinone in our case, could potentiate this effect, even in severe tricuspid infective endocarditis. Due to the rarity of tricuspid candidal infective endocarditis, regular follow-up is vital for patients with preexisting bioprosthetic valve replacement. Follow-up for these patients may allow providers to catch the symptoms of developing endocarditis and prevent disease progression. These appointments may also decrease other risk factors for the disease, including but not limited to infected lines.
